# Co-expression network of mRNA and DNA methylation in first-episode and drug-naive adolescents with major depressive disorder

**DOI:** 10.3389/fpsyt.2023.1065417

**Published:** 2023-02-23

**Authors:** Yuanmei Tao, Hang Zhang, Meijiang Jin, Hanmei Xu, Shoukang Zou, Fang Deng, Lijuan Huang, Hong Zhang, Xiaolan Wang, Xiaowei Tang, Zaiquan Dong, Yanping Wang, Li Yin

**Affiliations:** ^1^Department of Psychiatry, West China Hospital of Sichuan University, Chengdu, Sichuan, China; ^2^The Fourth People's Hospital of Chengdu, Chengdu, Sichuan, China; ^3^Institute for Systematic Genetics, Frontiers Science Center for Disease-Related Molecular Network, Chengdu, Sichuan, China; ^4^Sichuan Clinical Medical Research Center for Mental Disorder, Chengdu, Sichuan, China

**Keywords:** major depressive disorder, adolescent, mRNA, DNA methylation, co-expression network

## Abstract

**Objective:**

We explored the DNA methylation and messenger RNA (mRNA) co-expression network and hub genes in first-episode, drug-naive adolescents with major depressive disorder (MDD). To preliminarily explore whether adolescent MDD has unique mechanisms compared with adult MDD.

**Methods:**

We compared DNA methylation and mRNA profiles of peripheral blood mononuclear cells from four first-episode and drug-naive adolescents with MDD and five healthy adolescent controls (HCs). We performed differential expression analysis, constructed co-expression network, and screened the hub genes. And enrichment analysis was performed based on Gene Ontology (GO) and Kyoto Encyclopedia of Genes and Genomes (KEGG). We also downloaded DNA methylation and mRNA datasets of adults with MDD (GSE113725/GSE38206) from the GEO database, and performed differential expression and enrichment analysis.

**Results:**

Our clinical data showed that 3034 methylation sites and 4190 mRNAs were differentially expressed in first-episode, drug-naive adolescents MDD patients compared with HCs. 19 hub genes were screened out according to the high degree value in the co-expression network. The results from the GEO database showed that compared with adult HCs, there were 290 methylation sites and 127 mRNAs were differentially expressed in adult MDD patients.

**Conclusion:**

Compared with adolescent HCs and adult MDD patients, the DNA methylation and mRNA expression patterns of first-episode, drug-naive adolescent MDD patients were different. The co-expression network of DNA methylation and mRNA and the screened hub genes may play an important role in the pathogenesis of MDD in first-episode, drug-naive adolescents. Compared with adult MDD, adolescent MDD is more enriched in metabolism in terms of function and pathways.

## Introduction

Depression is one of the most common mental illnesses and is characterized by significant and long-lasting low mood, with a 4.4% lifetime prevalence globally (1). According to the results of a meta-analysis, the prevalence of depression in children and adolescents in China was 19.85% (95% confidence interval: 14.75%−24.96%) (2). At present, it is generally believed that depression is influenced by both genes and the environment, such as negative life events, including chronic diseases, unemployment, death of relatives, and violence (3). Through high-throughput sequencing technology, it was found that there were a large number of differentially expressed genes associated with interferon α/β signaling pathway between depression and healthy controls (4). At present, there have been meta-studies on exome sequencing and genome-wide association analysis (GWAS), which have found gene loci related to depression, and found that the genetic risk of depression was related to lower education and greater weight (5) and these genes were enriched in biological processes such as excitatory neurotransmission, mechanosensory behavior, post synapse, neuron spine and dendrite functions (6). Studies have also shown that the current findings of depression were mainly related to three biological processes: inflammation, glucocorticoid receptor function and neuroplasticity (7). According to the discovery of depression in the transcriptome, mRNA is gradually developing into molecular biomarkers, which are expected to be used in the diagnosis and treatment of many diseases (8).

Epigenetics refers to the influence on gene expression without changing the DNA sequence (9). With the development of the field of epigenetics, we have learned that through epigenetic changes, especially DNA methylation, the changes in gene activity established were the result of exposure to environmental adversity, social pressure, and traumatic experiences (10). At present, studies on depression have shown that the changes in methylation of some genes were closely related to depression, such as *BDNF* and *NR3C1* (11), and it has been found that the response of patients with major depressive disorder (MDD) to treatment was also related to methylation (12). Some studies have found that MDD patients with suicidal ideation, compared with MDD patients without suicidal ideation, *TPH2* mRNA and DNA methylation were significantly different (13).

We have not found any previous studies on the total potential landscape of mRNA and DNA methylation in adolescents with MDD. Therefore, we carried out this research, planning to obtain the differential expression of mRNA and DNA methylation in first-episode, drug-naive adolescent MDD patients and healthy controls. A co-expression network was conducted to obtain a more comprehensive, accurate picture of mRNA-DNA methylation interactions and obtained hub genes. Exploring the unique mechanism of MDD adolescents compared with MDD adults preliminarily.

## Methods

### Participants

This study recruited four first-episode, drug-naive adolescents with MDD from the Department of Psychiatry, West China Hospital, Sichuan University, from August to November 2019. Patients were diagnosed by two senior psychiatrists using the Chinese version of the Kiddie Schedule for Affective Disorders and Schizophrenia-Present and Lifetime Version (KSADS-PL) according to the “Diagnostic and Statistical Manual of Mental Disorders, Fourth Edition” (DSM-IV) (14). The Chinese version of the Beck Depression Inventory (BDI-II) was used to assess the severity of depressive symptoms, and patients were included when BDI ≥ 20 (15).

At the same time, five adolescent healthy controls were recruited from the community using posters. All healthy controls underwent neurological examinations and detailed semi-structured interviews to ensure that they had no history of mental illness or suicide attempts. MDD patients and healthy controls must be between 12 and 17 years old, right-handed, and must have at least completed primary education and can understand the content of the mental scale to be included. We used Hypomania Symptom Checklist-32 to exclude bipolar disorder and excluded subjects with alcohol or drug abuse, severe physical illness, or other axis I or II mental illness. All subjects and their guardians signed written informed consent. This study was approved by the Ethics Committee of West China Hospital of Sichuan University.

### RNA and DNA methylation extraction and sequencing

#### RNA

TRIzol (Invitrogen) reagent was used to extract total RNA from peripheral blood mononuclear cells. Only the ratio of 28S:18S in agarose gel electrophoresis was ≥1.5, and the RNA purity result detected by Nanodrop showed that the optical density ratio at 260 nm:280 nm was 1.8–2.2, and Qubit analysis of the concentration was ≥500 ng/μl. Follow-up analysis was carried out if the above conditions were met.

After the mRNA was enriched, it was randomly interrupted into short fragments of about 200bp and cDNA was synthesized by reverse transcription. Double strand cDNA was purified using AMPure XP beads. The terminal was then repaired and poly-A tail and sequencing splice were added. The USER enzyme is used to make it into a single-strand cDNA. The final cDNA library was obtained by PCR amplification. Bridge PCR amplification was performed on the cBot instrument to generate clusters. Sequencing was performed on the Illumina sequencing platform in 2 × 150 sequencing mode, and FastQC software (version 0.11.5) was used for quality control analysis of the preprocessed data. The average length of the sequence was about 149 bp after removing the low-quality fragments.

#### DNA methylation

Whole-genome DNA was extracted from peripheral blood mononuclear cells and treated with bisulfite to form transformed DNA. The DNA library was amplified by PCR, and then the matching reagent of the Illumina chip was used to fragment DNA to form short fragments of DNA, which were hybridized with the Illumina8x1 850K chip and scanned with the iScan system.

After loading the original EPIC idat file, some noncompliant probe sites were filtered out according to the principle that the probe methylation sites had a *p* > 0.01 (16). The BMIQ (beta mixture quantile dilation) method was used to standardize the beta value (17).

### Differential expression of mRNAs and DNA methylation positions between adolescent MDD patients and HCs

#### mRNA

The transcriptome in each sample was assembled using StringTie software (version 1.3.1c). The numbers of known gene sequences were counted by HTSeq software, and the expression level of transcripts was calculated by StringTie with the number of transcripts per kilobase per million fragments (FPKM). Calculate the *p* value of each mRNA and use the Benjamini and Hochberg correction multiple method for adjusted *p* value. The DESeq2 package of R (version 4.1.0) was used to screen the differentially expressed mRNAs between MDD patients and healthy controls based on the transcript expression level satisfying |log2FoldChange| > 1 and *p* < 0.05. If there is any duplicate of gene names associated with differential expressed transcripts, we do random deletion.

#### DNA methylation

The limma package of R was used to construct a linear model to calculate the p value of differentially methylated positions, and then the Benjamini and Hochberg method was used to perform multiple tests and calculate the adjusted *p* value. Screening of differentially methylated positions under the condition of *p* < 0.01. We randomly deleted the duplicate gene names.

Two-way hierarchical clustering of differential mRNAs and DNA methylation positions was performed. Differentially expressed mRNAs and DNA methylation positions were functionally enriched using Gene Ontology (GO) and Kyoto Genome and Genome Encyclopedia (KEGG). Fisher test was used to test the significance of enrichment.

### Validation of differentially expressed mRNAs

Four mRNAs were randomly selected from the differentially expressed mRNAs and verified by SYBR Green quantitative PCR (qRT-PCR) in the previous four MDD adolescents and five healthy controls. Total RNA was extracted with TRIzol, and reverse transcription was performed according to the instructions of the cDNA synthesis kit. β-actin was selected as the internal reference gene, and the primers used are shown in Table 1. Quantitative PCR was performed by Sangon Biotech (Shanghai, China) with 40 cycles of 95°C for 15 s, 60°C for 60 s, and 95°C for 15 s. Melting curve analysis was performed, and the 2^−ΔΔCt^ method was used to calculate the levels of mRNAs relative to β-actin levels. The experiment was carried out three times.

**Table 1:** Real-time fluorescence quantitative PCR primer sequences.

**mRNA**	**Direction**	**Primer sequences (5^′^-3^′^)**
*ASB13*	Forwards	TCAAGAATGTTGACCTCATCGA
	Reverse	AGTCAGAGGTGTCTTTTCGTAG
*LPIN1*	Forwards	GAGCAGCAGAACTCTTCCTAAT
	Reverse	CTTTTGCAATCTACCAGGCTAC
*PAPOLG*	Forwards	TCCACATCAACTCGAACAGTAA
	Reverse	GTTTGGACCAATCTGACTTTCC
*PPP1R16B*	Forwards	GATGAGATGCCAATAGACCTGT
	Reverse	GCTGTGACTTCATGATCACATC

### Co-expression network of mRNA and DNA methylation

Pearson analysis was used to identify the co-expression relationship between differentially expressed mRNAs and DNA methylation positions. An absolute value of Pearson correlation coefficient ≥0.95 was used to screen out target genes and construct a network of co-expression of mRNA and DNA methylation. Hub genes were screened out according to the high degree value in the co-expression network, and visualized by Cytoscape software (18).

### Analysis of data downloaded from the GEO database

#### mRNA

We selected a dataset of mRNA for patients with MDD in the GEO database: GSE38206. RNA was extracted from the subjects' peripheral blood mononuclear cells and analyzed using an Agilent 2100 Bioanalyzer (Agilent Technologies, Santa Clara, CA, USA). For the purpose of this study, we extracted clinical information and mRNA expression data from nine adult MDD patients and nine adult HCs. Then, we used the limma package in R to screen out the difference in mRNA between the two groups according to the criteria of |log2 (fold change)| >1 and *p* < 0.05. The ggplot2 package in R was used to draw a volcano plot and heatmap of differentially expressed mRNAs. Then we performed GO and KEGG enrichment analysis.

#### DNA methylation

The GSE113725 dataset downloaded from the GEO database contained the DNA methylation data of adult MDD patients and HCs. This experiment used a standard phenol-chloroform extraction method to isolate genomic DNA from whole blood, and the samples were processed in accordance with the Illumina Infinium HumanMethylation450K BeadChip (Illumina). For the purpose of this study, we extracted the clinical information data and DNA methylation expression data of 49 adult MDD patients and 48 adult HCs. We used the same method to process the methylation data and then analyzed the differential DNA methylation positions between the two groups according to *p* < 0.01. Then, we used ggplot2 to draw a volcano plot and heatmap of differentially expressed DNA methylation positions. Then GO and KEGG enrichment analysis were performed.

We searched for the intersection of differentially expressed mRNAs and DNA methylation positions between our clinical data and GEO database, and compared their GO and KEGG results.

## Results

### Analysis of adolescent MDD patients and HCs

**Figure 1:**
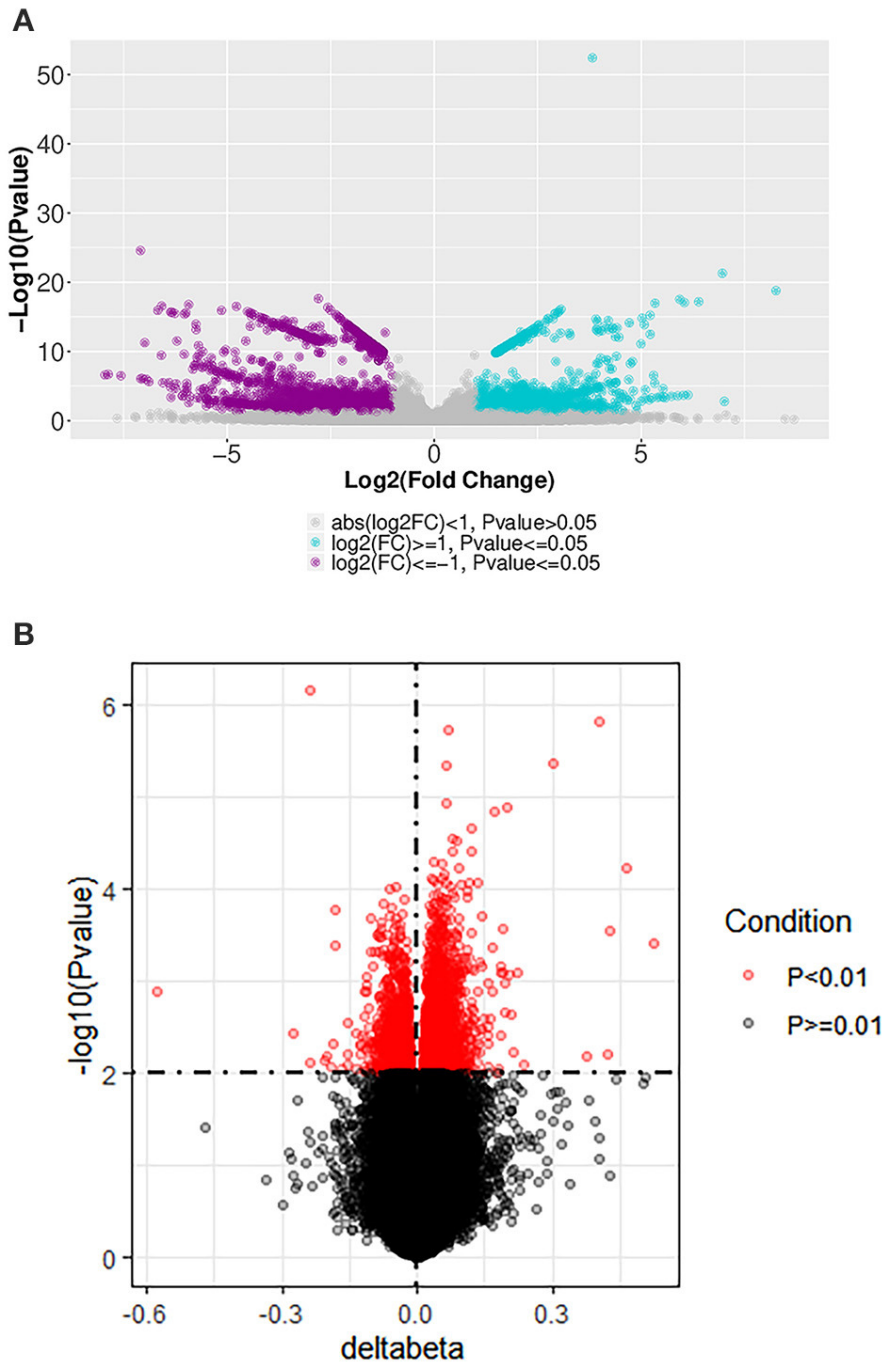
Volcano plot of differentially expressed mRNA and DNA methylation positions in first-episode, drug-naive adolescents with MDD. **(A)** mRNAs differentially expressed between first-episode, drug-naive adolescents with MDD and healthy controls. Blue and purple points indicate genes that were up- or down-regulated, respectively, more than two-fold in patients. **(B)** DNA methylation positions differentially expressed between first-episode, drug-naive adolescents with MDD and healthy controls.

**Figure 2:**
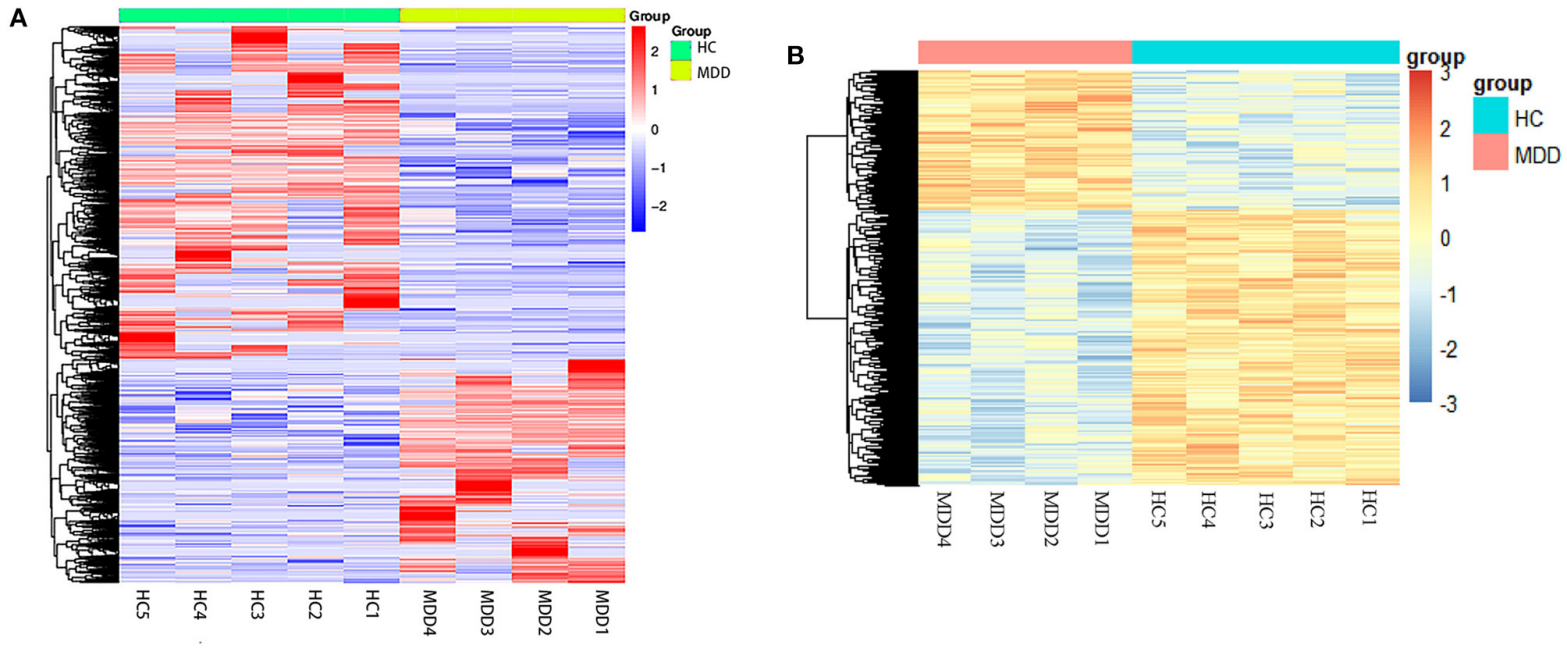
Heatmap of differentially expressed mRNA and DNA methylation positions in first-episode, drug-naive adolescents with MDD. Hierarchical clustering of mRNAs **(A)** and DNA methylation positions **(B)** between first-episode, drug-naive adolescents with MDD and healthy controls (HCs). The results showed different patterns between the two groups and homogeneity within each group. Red and blue indicate up- or down-regulation (hyper-methylated or hypo-methylated), respectively, in patients.

In our subjects for whole-genome methylation sequencing, the mean age of first-episode, drug-naive adolescent patients with MDD was 13.75 years old (SD: 1.50), and 100% were females. While the mean age of adolescent controls was 13.40 years old (SD: 0.55), and the female group accounted for 40%. We identified 4,190 mRNAs whose expression differed significantly between first-episode, drug-naive MDD patients and HCs. Of these mRNAs, 2,463 were down-regulated and 1,727 were up-regulated in patients (Figures 1A, 2A). The most up-regulated mRNA was *LPIN1* [log2(fold change) = 8.26, *p* =1.62E−19], and the most down-regulated mRNA was *PPP1R16B* [log2(fold change) = −7.95, *p* = 2.36E−07]. The top 20 differential mRNAs are listed in Table 2 (see Supplementary Table 1 for details).

**Table 2:** Top 20 differential expression mRNAs between first-episode, drug-naive adolescents with MDD and HCs.

**Symbol**	**Regulation**	**Log2 FoldChange**	***P* value**	**FDR**
*LPIN1*	Up	8.26	1.62E−19	4.97E−15
*PPP1R16B*	Down	−7.95	2.36E−07	3.69E−05
*PIK3R5*	Down	−7.87	1.91E−07	3.00E−05
*PAPOLG*	Down	−7.57	3.07E−07	4.75E−05
*GIGYF2*	Down	−7.09	2.55E−25	1.96E−20
*YAF2*	Down	−7.09	8.26E−07	1.24E−04
*HYOU1*	Down	−7.05	7.94E−07	1.19E−04
*HSPH1*	Up	7.01	1.57E−03	9.78E−02
*TRERF1*	Down	−6.99	5.26E−12	1.81E−09
*ASB13*	Up	6.96	4.97E−22	2.54E−17
*RAB1A*	Down	−6.89	2.27E−06	3.32E−04
*APLP2*	Down	−6.59	3.48E−10	5.97E−08
*SH3BP4*	Down	−6.58	2.63E−17	3.10E−13
*PLEKHO2*	Down	−6.55	3.13E−06	4.50E−04
*C4orf33*	Down	−6.41	2.96E−06	4.25E−04
*RGPD3*	Up	6.39	6.28E−18	1.21E−13
*DCTD*	Down	−6.36	1.36E−05	1.81E−03
*UBE2D3*	Down	−6.36	2.06E−16	1.31E−12
*SLC25A36*	Down	−6.35	4.02E−04	3.54E−02
*SULF2*	Down	−6.34	1.99E−16	1.31E−12

“Down” means that the mRNA was down-regulated in the MDD group compared with the HC group, while “Up” means that the mRNA was up-regulated. MDD, major depressive disorder; HC, healthy control. The top 20 were chosen based on the absolute value of the log2(fold change).

We found 3,034 DNA methylation positions that were differentially expressed in first-episode, drug-naive MDD patients and HCs. Compared with healthy controls, 1,028 positions were hypo-methylated, and 2,006 positions were hyper-methylated in patients (Figures 1B, 2B). The most significant hyper-methylated position was *REPIN1* (deltabeta = 0.52, *p* = 3.87E−04), and the most significant hypo-methylated position was *SLC36A3* (deltabeta = 0.58, *p* = 1.31E−03). The top 20 differential methylation positions are shown in Table 3 (see Supplementary Table 2 for details).

**Table 3:** Top 20 differential expression DNA methylation positions between first-episode, drug-naive adolescents with MDD and HCs.

**Gene_Name**	**Regulation**	**deltaBeta**	***p* value**
*SLC36A3*	Up	0.58	1.31E−03
*REPIN1*	Down	−0.52	3.87E−04
*NINJ2*	Down	−0.47	5.82E−05
*GPR88*	Down	−0.43	2.86E−04
*HLA-DRB1*	Down	−0.42	6.26E−03
*LOC100049716*	Down	−0.40	1.49E−06
*GSTM1*	Down	−0.38	6.56E−03
*ATP5S*	Down	−0.30	4.24E−06
*HS1BP3*	Up	0.28	3.63E−03
*PHGR1*	Down	−0.24	8.15E−03
*NDRG4*	Down	−0.22	7.90E−04
*ADCY9*	Down	−0.21	5.83E−03
*PPFIA4*	Up	0.21	7.03E−03
*FAM45A*	Down	−0.21	2.32E−03
*SLCO3A1*	Down	−0.21	8.34E−04
*GALNT2*	Down	−0.20	1.27E−05
*HLA-DQB2*	Up	0.20	6.48E−03
*PTEN*	Down	−0.20	2.15E−03
*ACSF3*	Down	−0.19	1.03E−03
*FOXN3*	Up	0.19	8.61E−03

“Down” means that the position was hypo-methylated in the MDD group compared with the HC group, while “Up” means that the position was hyper-methylated. MDD, major depressive disorder; HC, healthy control. The top 20 were chosen based on the absolute value of the deltaBeta.

**Figure 3:**
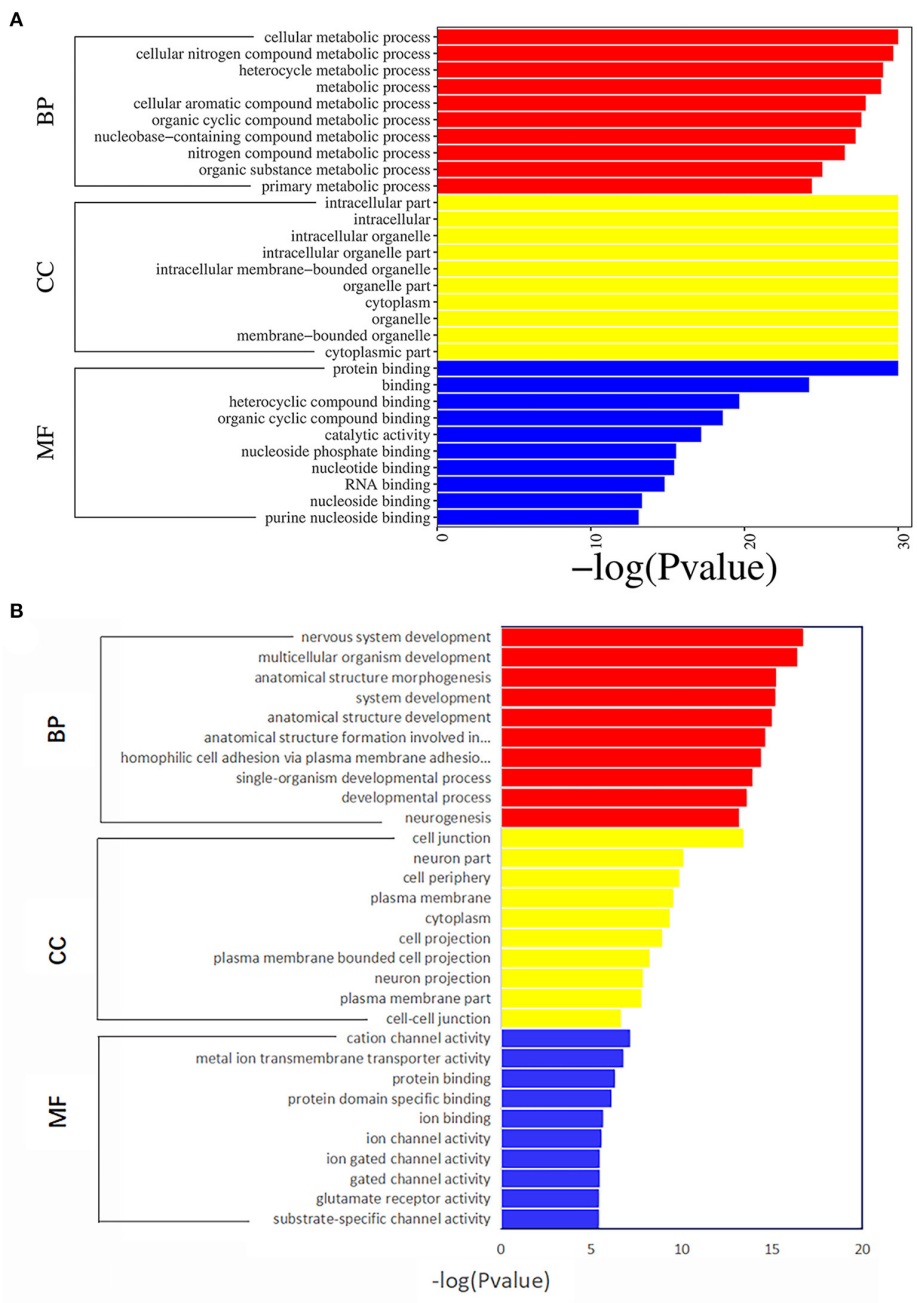
The top 10 enriched Gene Ontology terms for differentially expressed mRNAs **(A)** and DNA methylation positions **(B)** in first-episode, drug-naive adolescents with MDD. BP, biological processes; CC, cellular components; MF, molecular functions.

**Figure 4:**
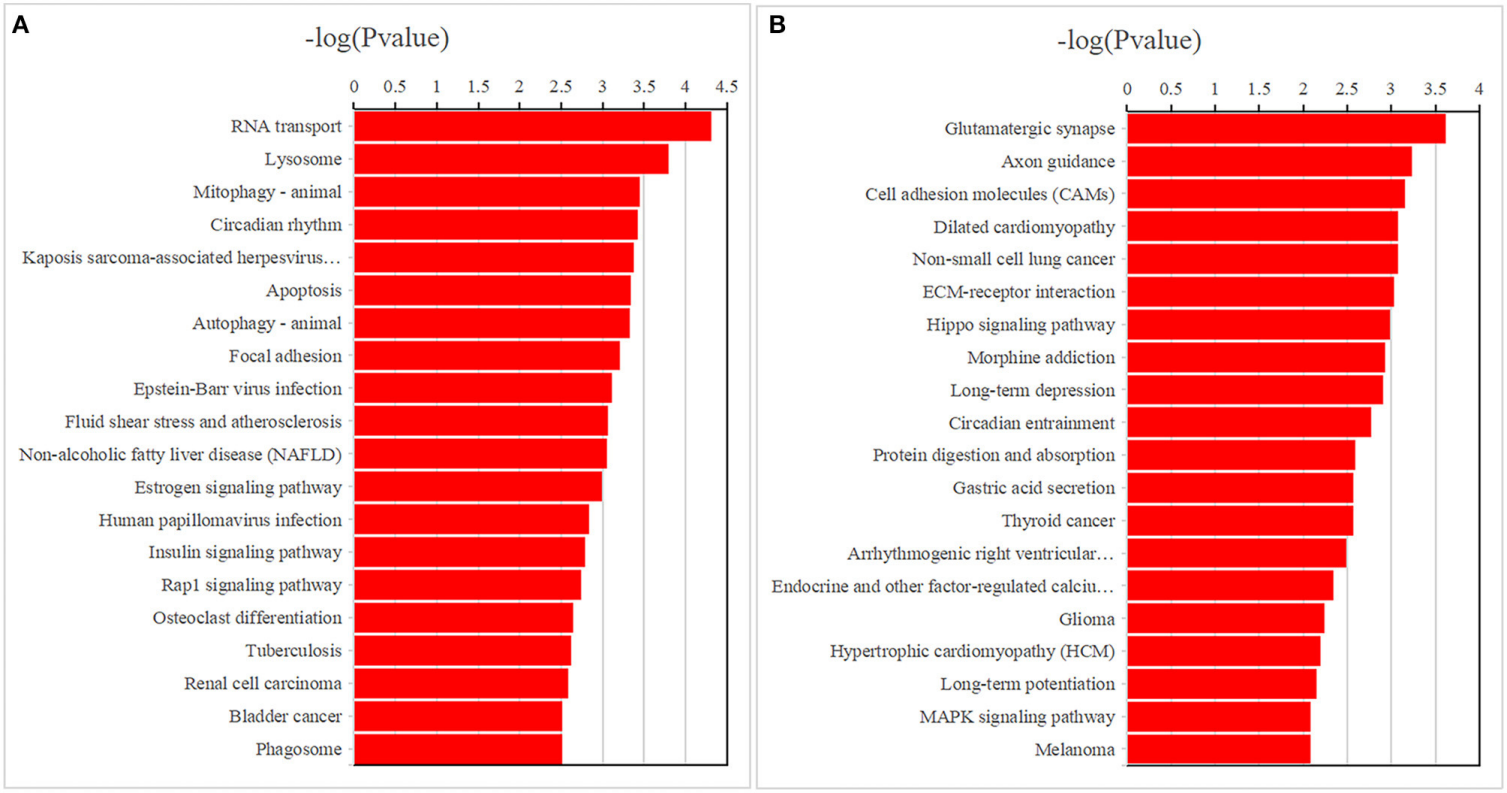
The top 20 enriched Kyoto Encyclopedia of Genes and Genomes pathways for differentially expressed mRNAs **(A)** and DNA methylation positions **(B)** in first-episode, drug-naive adolescents with MDD.

GO analysis results of the 4,190 mRNAs screened above showed that they were mainly enriched in 1,645 biological processes, 307 cell components and 365 molecular functions (Figure 3A; Supplementary Table 3). KEGG analysis showed that differential mRNAs were mainly enriched in pathways related to RNA transport, lysosome, mitophagy-animal, circadian rhythm, kaposis sarcoma-associated herpesvirus infection, apoptosis, autophagy-animal, and focal adhesion (Figure 4A; Supplementary Table 4).

The GO analysis of the differentially methylated positions found that they were mainly enriched in 1,108 biological processes, 205 cell components and 216 molecular functions (Figure 3B; Supplementary Table 5). KEGG analysis found they were mainly enriched in glutamatergic synapse, axon guidance, cell adhesion molecules, dilated cardiomyopathy, non-small-cell lung cancer, ECM-receptor interaction and other signaling pathways (Figure 4B; Supplementary Table 6).

To verify the differentially expressed mRNAs, we selected 4 mRNAs (2 up-regulated, 2 down-regulated). The quantitative PCR results were consistent with our RNA sequencing results (Table 4), which indicates that the results of RNA-seq were reliable.

**Table 4:** qPCR results verified the differentially expressed mRNAs between first-episode, drug-naive MDD patients and healthy controls.

**mRNA**	**Sequencing**	**qPCR**
*ASB13*	6.96	0.21
*LPIN1*	8.26	3.05
*PAPOLG*	−7.57	−0.44
*PPP1R16B*	−7.95	−0.34

Value: the log2 (fold change) of differentially expressed.

**Figure 5:**
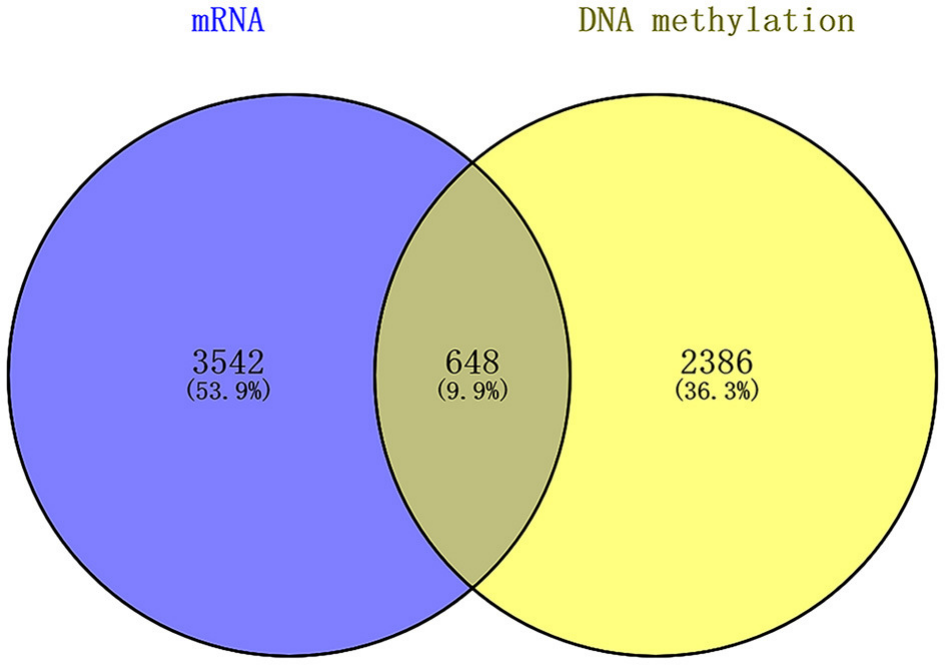
Overlap between the differentially expressed mRNAs and DNA methylation positions between first-episode, drug-naive adolescents with MDD and healthy controls.

We found that there were 648 genes overlapping in the differentially expressed mRNAs and DNA methylation positions, which are displayed by a Venn diagram (Figure 5).

**Figure 6:**
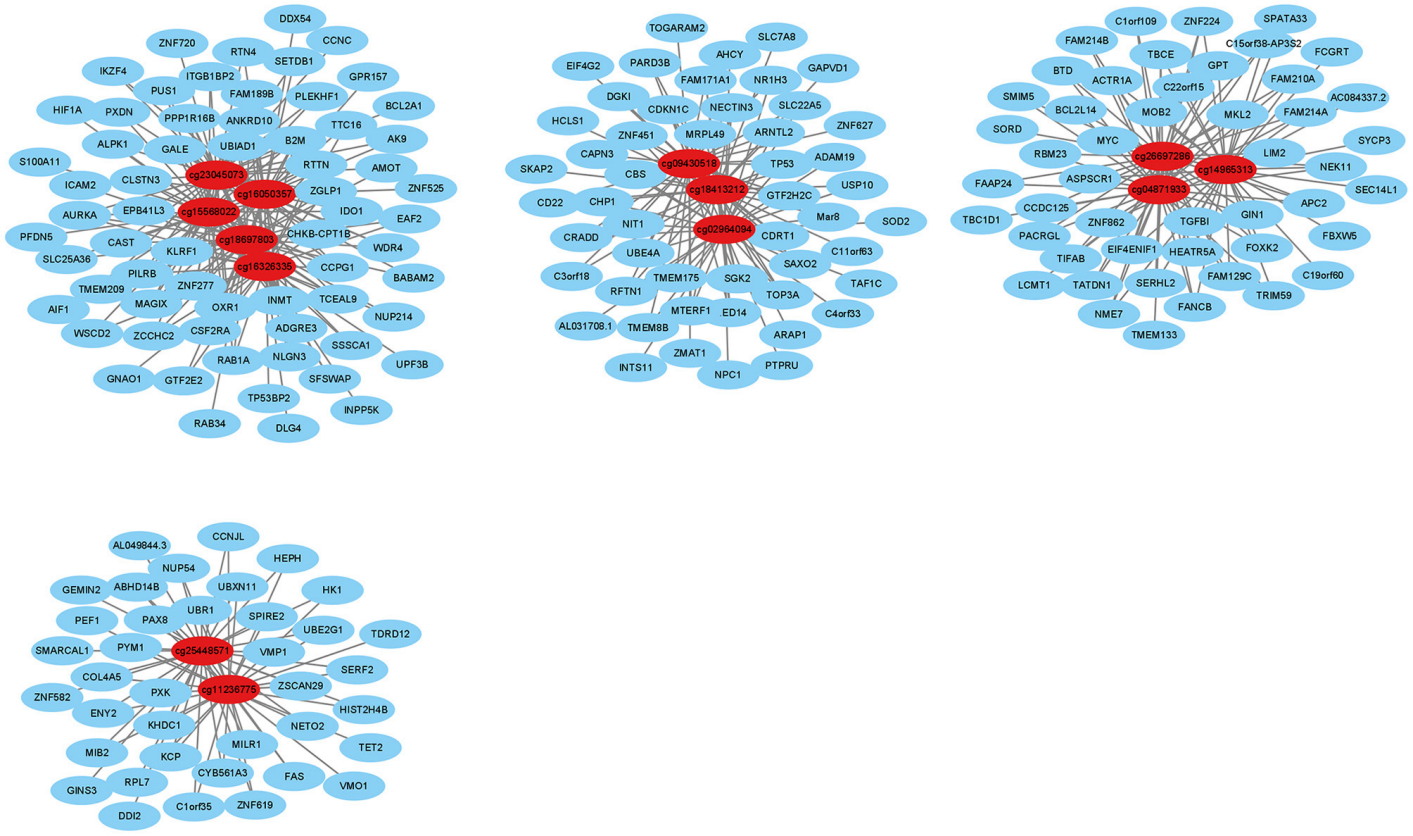
Interactions between differentially expressed DNA methylation positions and mRNAs. The red dots indicate DNA methylation positions, and the blue dots indicate mRNAs.

We identified 20,623 pairs of related DNA methylation positions and mRNAs, from which we generated a co-expression network based on the top 13 degrees with a minimum degree of 30 (Figure 6). In the network, the relationship of mRNA and DNA methylation was not a simple one-to-one relationship. From this, we could see that one DNA methylation position could regulate multiple mRNAs, and the same mRNA was regulated by multiple DNA methylation positions. According to the high degree value in the co-expression network, the top 41 degrees with a minimum degree of 25 (the top 5.8%) were used as screening conditions to obtain the hub genes. Then, taking the differentially methylated positions into consideration, 19 hub genes were finally screened out (Table 5).

**Table 5:** Information on 19 hub genes.

**Probe ID**	**Name**	***p* value**
cg02625318	*SLC6A6*	7.32E−03
cg18697803	*SCN10A*	8.98E−03
cg23045073	*NPHS2*	4.10E−03
cg14128973	*CNN1*	6.90E−04
cg02964094	*CC2D2A*	1.79E−03
cg13956151	*ZC3H7B*	6.16E−03
cg15568022	*C4orf3*	2.42E−03
cg09940915	*EPHA8*	6.04E−04
cg09560953	*UBE2E1*	3.34E−03
cg09293560	*REPIN1*	3.87E−04
cg18158149	*NOS1AP*	2.29E−03
cg18202492	*TMEM48*	9.85E−03
cg18413212	*GULP1*	5.07E−03
cg16050357	*TEX14*	6.28E−03
cg21385629	*PARD6G*	7.15E−03
cg01422797	*PRDM13*	2.86E−03
cg04075012	*ATG12*	7.02E−03
cg13696752	*C9orf68*	4.04E−03
cg20485733	*RAP1GAP2*	4.76E−03

### Analysis results of the downloaded data from the GEO database

The demographic information of mRNA data downloaded from GEO database was as follows: the average age of the control group was 53.78 years (SD: 9.22), and females accounted for 55.56%. While the patient group was 57.22 years (SD: 8.48), and females accounted for 55.56%. Compared with control group, 33 mRNAs were up-regulated and 94 mRNAs were down-regulated in patient group (Supplementary Table 7). These differential mRNAs are shown by a volcano plot and a heatmap (Supplementary Figure 1). GO analysis of differentially expressed mRNAs found that they were mainly enriched in 139 biological processes, 9 cell components and 15 molecular functions (Supplementary Table 8). KEGG analysis found that they were enriched in the AGE-RAGE signaling pathway in diabetic complications, coronavirus disease-COVID-19, rheumatoid arthritis, IL-17 signaling pathway, staphylococcus aureus infection, viral protein interaction with cytokine and cytokine receptor, TNF signaling pathway and other pathways (Supplementary Table 9).

The demographic information of DNA methylation data downloaded from GEO database was as follows: The average age of the control group was 45.50 years (SD: 9.99), and females accounted for 75.00%. While the patient group was 45.96 years (SD: 9.36), and females accounted for 73.47%. We found 288 differentially expressed DNA methylation positions, including 137 hyper-methylated positions and 151 hypo-methylated positions in adult depression patients (Supplementary Figure 2; Supplementary Table 10). We found that differentially expressed methylation positions were mainly enriched in 83 biological processes, 16 cell components and 24 molecular functions (Supplementary Table 11). KEGG results showed that they were mainly enriched in peroxisome, wnt signaling pathway, taurine and hypotaurine metabolism, primary bile acid biosynthesis, 2-oxocarboxylic acid metabolism, nucleocytoplasmic transport, ribosome biogenesis in eukaryotes and other pathways (Supplementary Table 12).

### Overlap between the results of adolescent MDD and adult MDD

Intersecting the differentially methylated positions analyzed on GEO with the 648 genes we obtained from adolescents with MDD, we found a total of 12 results: *PLEKHA7, SDR39U1, DIP2C, VEPH1, TBC1D14, ZNF839, SLC25A29, IPO7, RBM15, MAP2K5, PPM1D, UBAC2*, but no results for mRNAs. We searched for the KEGG information of the 12 genes on the KEGG webpage (https://www.kegg.jp/) and found only two pathways information for *IPO7*: nucleocytoplasmic transport and MAPK signaling pathway; 5 pathways information for *MAP2K5*: MAPK signaling pathway, gap junction, neurotrophin signaling pathway, oxytocin signaling pathway, fluid shear stress and atherosclerosis; and one pathway information for *PPM1D*: p53 signaling pathway.

## Discussion

The study found that compared with healthy adolescents, first-episode, drug-naive adolescents with MDD have a large number of differentially expressed mRNAs and DNA methylation positions. Some of the differentially expressed mRNAs found in first-episode, drug-naive adolescents with MDD were also found in previous genome-wide analyses of untreated adults with MDD (19). Some have also been found in a depression-related immune studies, such as *IL4, IL10, IL18, CSF2R, IFNGR1, CCL2, LTA, PRKCSH, PSMD13, IDO1, FKBP4* (20), which echoed the acute immune inflammatory response immediately after electroconvulsive treatment in patients with depression (21), indicating that immunity may play an important role in patients with depression. Previous studies have found that the mRNA levels of *HDAC5* and *CREB* in patients with depression were significantly higher than those in the control group, and their mRNA levels decreased after antidepressant treatment (22), indicating the importance of these two genes in depression. Regarding the differentially methylated positions found in first-episode, drug-naive adolescents with MDD, studies have found that the methylation of genes such as *ACIN1, ACTR1A, ADCY7, ADCY9, ADD1, AMICA1, ANGPT2, ANK1, ANXA3, AP2A2, AP2B1, AP3B1, APP, ARIH2, ARTN, ATP1B1* and *BAIAP2* in patients with depression is involved in the immune process, indicating an interaction between depression and inflammation-related diseases (23). Some studies have shown that the DNA methylation of *BDNF* mediates the association between childhood trauma and depression to a certain extent (24), and can be used as an effective diagnostic biomarker for MMD (25). And one study found that higher *BDNF* was significantly associated with suicidal ideation (26).

The co-expression network we constructed showed that the relationship between mRNA and DNA methylation was not a simple 1:1 pairing. Through the co-expression network, we obtained 19 hub genes. Among them, *SCL6A6* is a taurine transporter TauT. In a study of mouse hippocampal slices, it was found that the accumulation of some amino acids, including taurine, in cells can increase synaptic potential (27), and studies have also found that the transport process of guanidine compounds in blood cerebrospinal fluid involved in *SLC6A6* may be related to neurological disorders in the brain (28). *SCN10A* is a voltage-gated sodium channel, and many studies on pain have shown that it is closely related to mechanical pain (29). Interestingly, menthol, a natural compound of plant origin, acts as an analgesic by inhibiting the activation of Na ion channels (30), and most current studies have shown that depression and pain symptoms usually coexist and share biological pathways and neurotransmitters (31). In a meta-analysis of a genome-wide association study of depression, it was found that the differential expression of *ZC3H7B* in three included studies was highly consistent (32), indicating that this gene may be related to depression. Studies have found that the *EPHA8* receptor (a member of the Eph family of tyrosine protein kinase receptors) is involved in axonal pathfinding (33) and cell contact-dependent signaling in axon growth and guidance during the development of the mammalian nervous system (34). Studies have shown that *REPIN1* gene variation affects the expression of *REPIN1* target gene (including glucose and fatty acid) transporters to varying degrees and plays a role in glucose and lipid metabolism (35), and we found in our research that differentially expressed mRNAs and DNA methylation positions were enriched in the process of sugar and lipid metabolism. Previous studies have found that the *NOS1AP* SNP rs386231 was associated with an increase in the severity of depression (36), and in mouse model experiments, it has been found that the decline in viral-mediated *CAPON* (carboxy-terminal PDZ ligand of NOS1) in the inner prefrontal cortex significantly reversed the chronic and unpredictable depression-like behavior of mildly stressed mice (37). The *ATG12* system is one of the main systems for the formation and maturation of autophagosomes (38), and studies have shown that autophagy is involved in the pathophysiology and treatment of MDD (39).

In the overlapping results of our first-episode, drug-naive adolescents with MDD and GEO data, *DIP2C* has been proven to play an important role in brain development and function (40), and studies have found that when the maternal circadian rhythm was disturbed, the expression of *DIP2C* methylation in the placenta was up-regulated, which might have an impact on the fetus (41). *VEPH1* is related to brain development and the thickness of the transverse temporal gyrus (42). Studies have shown that *TBC1D14* is related to the autophagy process (43), which echoed our enrichment of differentially expressed mRNAs in the autophagy pathway. *IPO7* is essential for the function of glucocorticoids, and the expression of its cofactor RanGTP (Ras protein GTP) is reduced during the process of glucocorticoid insensitivity induced by oxidative stress (44). Previous studies have found that *MAP2K5* mRNA and its predicted miRNA are potentially important contributors to depression and anxiety-related characteristics (45). The lack of *PPM1D* in the mouse model increased the release of inflammatory cytokines in the brain, increased the number of activated microglia and macrophages in the brain, aggravated brain tissue lesions, and aggravated cognitive impairment, which proved that it was a key neuroprotective agent to prevent brain damage caused by a hypoxic inflammatory response (46).

This study found that differentially expressed mRNAs and DNA methylation are enriched in many biological processes. Some of these have also been found in previous studies on the pathogenesis of MDD, such as cellular response to lipopolysaccharide, biotic stimulus, and molecules of bacterial origin, negative regulation of multi-organism process, response to temperature stimulus, unfolded protein binding, collagen binding and other processes (47). In a previous study on genome-wide analysis of blood for depression, some similar mechanisms that might be related to depression have also been discovered: positive regulation of biosynthesis, RNA metabolism, gene expression, macromolecule metabolism and nitrogen compound metabolism processes (19).

This study found that KEGG enrichment in first-episode, drug-naive adolescents with MDD was not only enriched in the well-recognized dopaminergic synapse, serotonergic synapse, and glutamatergic synapse pathways but also enriched in substance addiction-related pathways, such as cocaine addiction, amphetamine addiction, nicotine addiction, and morphine addiction, and some signal transduction pathways, such as the cAMP signaling pathway and taste transduction, and enriched in some rhythm-related pathways, such as circadian rhythm and circadian entrainment (48). A MDD mouse model suggested that retrograde endocannabinoid signaling might be a potential mechanism for antidepressant effects (49), and a systematic review of articles concluded that ketamine had a rapid antidepressant response through GABAergic synapses (50).

Growth- and proliferation-related enrichment results were found in both adolescent and adult MDD patients, such as: mitogen-activated protein kinase (MAPK) pathway, phosphatidylinositol 3'-kinase (PI3K)-Akt pathway, mammalian target of rapamycin (mTOR) pathway, mitotic cell cycle process, developmental cell growth, and positive regulation of developmental growth. For example, in the MAPK signaling pathway, mouse model studies have shown that compared with depressed mice, the hippocampus of wild-type mice had higher expression of p-JNK and p-p38 proteins, which indicated that the mitochondrial activated protein kinase (MAPK) pathway was activated, and further research on inflammatory signaling pathways may have a bright future in the prevention and treatment of depression (51). A systematic review article showed that in humans and various animal models of chronic depression, the ERK signal of the extracellular signal-regulated kinase of MAPK was significantly reduced in the prefrontal cortex and hippocampus. These two core areas are related to depression, and direct upstream regulators of ERK and MAPK phosphatase (MKP) also play an important role in antidepressant therapy (52). According to the comparison of enrichment results between adolescents and adults with MDD, we found that the results of MDD adolescents were more enriched in synthesis and metabolic processes of cellular nitrogen compound, heterocycle metabolic process, cellular aromatic compound, organic cyclic compound, organic substance, nucleobase-containing compound, etc. While MDD adults were more enriched in immune and infection-related processes, such as: regulation of phagocytosis, leukocyte chemotaxis, monocyte chemotaxis, ERK1 and ERK2 cascade, and defense response to virus by host.

Compared with GEO data, we found more differentially expressed DNA methylation positions and mRNAs in our clinical data. This was mainly due to the rapid development of chip technology. We used the Illumina Infinium HumanMethylation850K BeadChip, while the download data used the Illumina Infinium HumanMethylation450K BeadChip. Another reason for the difference was that our research subjects were adolescents with a small number of subjects, while the research subjects in the GEO database were middle-aged people with a large number of subjects. Unfortunately, we were not able to find the complete pathway information of the overlapping results found in our clinical dataset and the GEO download dataset on the KEGG webpage, and we were not able to obtain their GO analysis results. And we did not find suitable mRNA and methylation data for children and adolescents with depression in the GEO database for analysis. This paper was only a preliminary analysis of the mRNA and DNA methylation of adolescents with MDD, and there might be greater heterogeneity in the case of a small number of people. Therefore, we selected first-episode, drug-naive adolescents with major depressive disorder to minimize heterogeneity. Age and education years matched between the two groups. However, gender differences did exist between the two groups, which was the weakness of this paper. This paper was the first to explore methylation differences in adolescent depression, and these influencing factors must be controlled for in future large sample studies.

In summary, our research results showed that there were many differentially expressed mRNAs and DNA methylation positions in first-episode, drug-naive adolescents with MDD, and they participated in multiple biological processes and signaling pathways and jointly participated in the occurrence and development of MDD. Both adolescent MDD and adult MDD found abnormal expression of the following genes: *PLEKHA7, SDR39U1, DIP2C, VEPH1, TBC1D14, ZNF839, SLC25A29, IPO7, RBM15, MAP2K5, PPM1D*, and *UBAC2*, and some of their KEGG pathway information was found: nucleocytoplasmic transport, MAPK signaling pathway, gap junction, neurotrophin signaling pathway, oxytocin signaling pathway, fluid shear stress and atherosclerosis, p53 signaling pathway. These genes and related pathways may have a strong relationship with depression. According to the comparison of enrichment results between adolescents and adults with MDD, we found that the results of MDD adolescents were more enriched in synthesis and metabolic processes, while MDD adults were more enriched in immune and infection-related processes. With the rapid development of sequencing technology, there has been much research on the genetics and epigenetics of depression, and some important discoveries have been made. However, we still found a lack of research on depression in children and adolescents. With the increasing incidence of depression in children and adolescents, we should pay more attention to this population.

## Data availability statement

The datasets presented in this study can be found in online repositories. The names of the repository/repositories and accession number(s) can be found in the article/Supplementary material.

## Ethics statement

This study was approved by the Ethics Committee of West China Hospital of Sichuan University. Written informed consent to participate in this study was provided by the participants' legal guardian/next of kin.

## Author contributions

Methodology and original draft preparation: LY and YT. Data collection: HaZ, MJ, HX, YT, SZ, FD, LH, HoZ, XW, XT, ZD, and YW. Project administration and funding acquisition: LY. All authors contributed to the article and approved the submitted version.
