# Supplementary material for: Co-expression network of mRNA and DNA methylation in first-episode and drug-naive adolescents with major depressive disorder
Source: Front Psychiatry. 2023 Feb 23;14:1065417. doi: 10.3389/fpsyt.2023.1065417 (PMC9995926; doi:10.3389/fpsyt.2023.1065417)
Supplement: Supplementary file 1 [file Data_Sheet_1.docx]

Supplementary figure 1. Differential analysis of mRNA data downloaded from the GEO database

Supplementary figure 1A Supplementary figure 1B


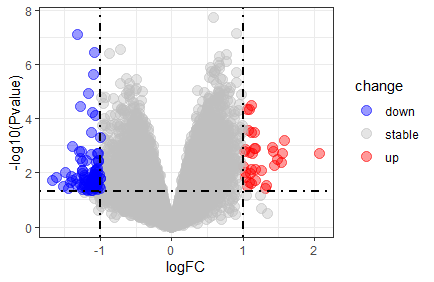

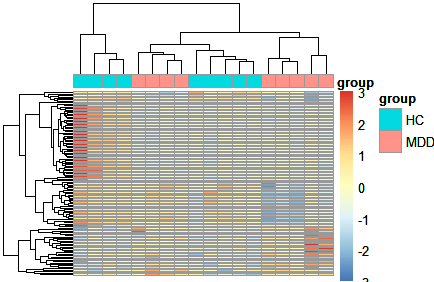


Supplementary figure 1A: mRNAs differentially expressed between adult patients with major depressive disorder and healthy controls. Supplementary figure 1B: Hierarchical clustering of mRNAs between adult patients with major depressive disorder and healthy controls. The results show different patterns between the two groups and homogeneity within each group. Red and blue of these two graphs indicate up- or down-regulation, respectively, in patients.

Supplementary figure 2. Differential analysis of DNA methylation data downloaded from the GEO database

Supplementary figure 2A Supplementary figure 2B


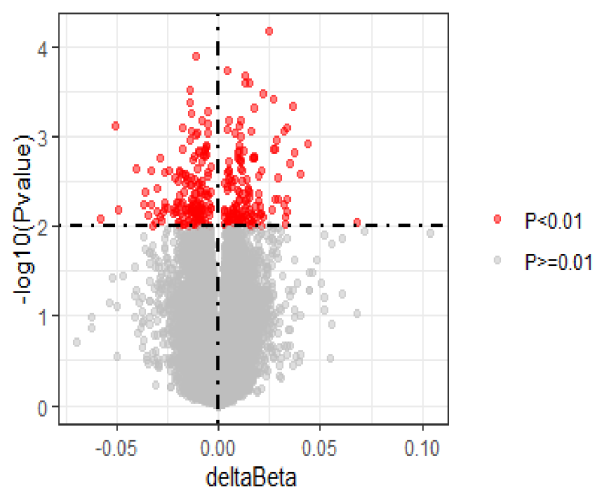




Supplementary figure 2A: DNA methylation positions differentially expressed between adult patients with major depressive disorder and healthy controls. Red points indicate genes that were differentially expressed DNA methylation positions. Supplementary figure 2B: Hierarchical clustering of differentially expressed DNA methylation positions between adult patients with major depressive disorder and healthy controls. The results showed different patterns between the two groups and homogeneity within each group. Red and blue indicate hyper-methylated or hypo-methylated, respectively, in patients.
